# Characteristic Processes in Close Peer Friendships of Preterm Infants at Age 12

**DOI:** 10.6064/2012/657923

**Published:** 2012-07-22

**Authors:** Mary C. Sullivan, Suzy Barcelos Winchester, Jeffrey G. Parker, Amy K. Marks

**Affiliations:** ^1^College of Nursing, The University of Rhode Island, Kingston, RI 02881-2021, USA; ^2^Brown Center for the Study of Children at Risk, Women & Infants Hospital, Providence, RI 02908, USA; ^3^Department of Psychology, University of Alabama, Tuscaloosa, AL 35487-0348, USA; ^4^Department of Psychology, Suffolk University, Boston, MA 02108, USA

## Abstract

Close friendships become important at middle-school age and are unexplored in adolescents born prematurely. The study aimed to characterize friendship behaviors of formerly preterm infants at age 12 and explore similarities and differences between preterm and full-term peers on dyadic friendship types. From the full sample of *N* = 186, one hundred sixty-six 12-year-old adolescents (40 born full term, 126 born preterm) invited a close friend to a 1.5 hour videotaped laboratory play session. Twenty adolescents were unable to participate due to scheduling conflicts or developmental disability. Characteristic friendship behaviors were identified by Q-sort followed by Q-factoring analysis. Friendship duration, age, and contact differed between the full-term and preterm groups but friendship activities, behaviors, and quality were similar despite school service use. Three Q-factors, leadership, distancing, and mutual playfulness, were most characteristic of all dyads, regardless of prematurity. These prospective, longitudinal findings demonstrate diminished prematurity effects at adolescence in peer friendship behavior and reveal interpersonal dyadic processes that are important to peer group affiliation and other areas of competence.

## 1. Introduction

Friendships become especially important in middle childhood as more time is spent with peers [1–3]. Close friendships, a mutual relationship with a same sex peer, develop gradually through middle childhood and adolescence, and influence both positive and negative social behaviors [4]. A close friendship-type is marked by interpersonal intimacy, sensitivity to the other, and efforts to make mutual interactions satisfying. These characteristic “friendship-type” social behaviors learned within the context of a close friendship may influence how the child processes, interprets, and responds to social behaviors [5]. High-quality friendships can foster positive self-esteem, social and emotional adjustment, and cognitive growth [6–8]. Past studies of friendship have primarily focused on healthy, normal developing children. Follow-up research of preterm infants has largely focused on cognitive outcomes, with limited attention to the social competence including peer friendship. In a US longitudinal study of early intervention effectiveness, early correlates of school-age behavior and social skills were examined in a subset of very high-risk preterm infants [9]. The children, who as infants suffered neonatal illnesses and whose parent was stressed, had lower social skills reported by parents and teachers at age 7.5 years. A possible explanation is that children born prematurely may have difficulty establishing and sustaining peer relationships because of a developmental delay or limited experiences with peers due to fragile health. Another explanation is posited from the neonatal period when the infant is medically fragile, maybe temperamentally difficult and parents are stressed, thus setting up lower reciprocity in parent-infant interaction leading to a problematic relationship [10].

The formation of friendships for children born preterm may be more challenging and is a developmentally necessary task that functions as a stepping stone to other developmental outcomes [11]. If social skills are underdeveloped in childhood and adolescence, there is reduced access to enriching social and learning experiences. Later, in early adulthood, these behaviors and skills are critical to community living and job skills. Therefore, this study aims to examine the dyadic processes between two middle school-age friends, one of whom is prematurely born, as they reach early adolescence where close friendships take center stage.

## 2. Dyadic Processes in Friendship

The focus of the present study is on the dyadic interaction between two close friends. Unlike peer acquaintances, the reciprocity between two close friends allows trust, self-disclosure, and testing of the other [3]. As such, observation of dyadic friendship processes allows a view of interpersonal processes such as leadership/dominance, synchrony, activity levels, cohesion, intimacy [2], information exchange, problem solving, conflict resolution, and self-exploration [3]. In early adolescence, friendship processes focus on support or acceptance by peers while simultaneously minimizing rejection [3]. These processes exhibited in close friendships afford the opportunity to practice social skills which are associated with later social adjustment and other domains of developmental competence [11].

## 3. Prematurity and Friendship

Early adolescence is a period in development when a child is beginning to have extensive changes in every aspect of individual development, especially the social domain as this is a time of intense sociability. To date, the studies investigating dyadic friendship processes have predominantly included physically healthy children. The preterm infant birth rate has risen steadily, until recently. Current US estimates are that 1 in 8 infants are born too early [16]. Follow-up reports suggest that infants born preterm often have subtle developmental challenges which extend into adolescence. These developmental challenges include delays in social skills, problem behaviors, poor academic achievement, and school-related resources which may hinder the formation of close friendships [9, 17–21]. The influence of prematurity combined with the demands of early adolescent development may challenge a formerly preterm child's ability for successful social relationships. In addition, sequelae of prematurity place them at risk for poor social functioning with peers. For example, children born preterm who have poor social skills in middle childhood may misinterpret peer behavior during a social exchange which results in an inappropriate response. Therefore, the complexity of verbal encoding and interpretation of another, which is necessary for successful social interaction, may be affected in children born preterm [22, 23].

Prior to adolescence, parents report that their prematurely born children have social difficulties and children describe their relationships as more difficult than children born at term with normal birth weight [10, 24]. In one study, 7-year-old children born <29 weeks of gestation experienced more verbal victimization (name-calling, teasing) by their peers even when children with motor, cognitive, and sensory deficits were excluded [23]. These findings were corroborated in a separate report of 10-year-old children born preterm (<1500 g) with or without cerebral palsy who experienced more physical and verbal victimization, and social isolation than full term peers [8, 23]. One plausible explanation for these findings includes the following: children born preterm have more health sequelae than their peers [25, 26]. Visual motor, minor motor problems, and subtle neurological deficits persist throughout childhood so they may appear clumsy to peers [19, 27] which may lead to victimization [8, 28].

At later ages, adolescents who were prematurely born rated themselves lower in romantic appeal, as well as scholastic, athletic, and job competence, and had difficulty making social contacts [29, 30]. Formerly preterm infants are reported to have more hyperactivity, behavioral problems, and anxiety compared to full term peers from adolescence to young adulthood [31, 32]. These challenges may further complicate or interfere with successful social interactions. The processes underlying friendship have features that are common to larger social organizations such as school classes and teams. The present study with formerly preterm infants at age 12 may reveal that difficulties in social skills may affect friendship behaviors.

A social-cognitive-processing perspective was taken in this observational study. In a social interaction, each friend is required to perceive and respond to the other. This approach observes each child in the dyad to encode, interpret feedback and apply social-behavioral information within a social exchange [2]. We elected to focus on the interactive processes between two friends, one born prematurely.

The body of literature on children born preterm includes performance on social skills, not on friendship outcomes. Due to the dearth of literature on peer relationships with children born preterm, we proposed three exploratory goals. The first goal was to describe the demographic characteristics of close friends at age 12, one of whom was born prematurely. The second goal was to characterize their dyadic friendship behaviors and to examine concurrent validity of the dyadic friendship behaviors with independently coded dyad ratings. The third goal was to determine whether prematurity and gender differed between children born preterm and full term peers on friendship behaviors and the friend's perspective of the quality of the peer relationship.

Based on evidence of parent-infant stress in early infancy, with lingering subtle neurodevelopmental impairments which predispose adolescents to developmental immaturity and difficulty interpreting interaction processes [22], we hypothesized a difference in friendship behaviors between a prematurely born group and a full term group. We expected that children who were born prematurely would demonstrate subservient and timid behaviors in dyadic play due to inaccurate processing of social behavioral information, and likely follow the lead of their friend rather than show assertiveness. Since, the invited friend was identified as a close friend, we expected to see a moderate level of intimacy and self-disclosure in dyad pairs. We expected differences based on gender where girls would demonstrate more behaviors of intimacy and self-disclosure in dyadic interaction when compared to boys [13, 33].

## 4. Methods

### 4.1. Sample

This was a prospective, longitudinal design with infants born preterm recruited at birth and followed to age 12. The criteria for recruitment were prematurity (<37 weeks of gestation) and birth weight <1850 g. Medically, the preterm group included low-risk infants without neonatal complications, and high-risk infants who had one or more complications of prematurity including bronchopulmonary dysplasia, sepsis, necrotizing enterocolitis, meningitis, and intraventricular hemorrhage. A group of full term, healthy infants was recruited at the same time. The maternal criteria for infant recruitment were absence of maternal mental illness or intellectual disability, maternal age ≥16 years, and English as a primary language. Two hundred and thirteen infants were recruited shortly after birth at a major center with a level III Neonatal Intensive Care (NICU). At age 12, 87% (*N* = 186) of the original sample participated. There were no differences in neonatal illness, socioeconomic status, parent education, occupation, marital status, age, or race between those who participated at age 12 and the original birth sample.

### 4.2. Procedure

The children, their parent(s), and friend were seen in the hospital research laboratory at age 12. The study was approved by university and hospital institutional review boards. Study participants, their parents, and friend were fully informed of the study procedure. Informed consent was signed by the parent(s) and assent completed by study participant and friend.

The study participant or “target child” was asked to bring a close friend to the hospital laboratory for a 1.5-hour-play session. Each child separately completed the Friend Demographics and the Friendship Contact Checklist. The invited friend completed the Friendship Quality Questionnaire. They were reunited in the central assessment room for five play activities presented in a standard sequence. Except for the brief period when the examiner introduced each new activity, no study personnel were present in the room. The room was arranged comfortably with a couch, two beanbag chairs, and a table. The entire sequence was videotaped through a one-way mirror with microphones concealed in the ceiling. Varying incentive conditions were incorporated for each player for each activity. A pegboard was used to keep the running point total for each player and the examiner called attention to each player's score at the start of each new activity. The target child and friend were each given $15 as an incentive thank you for participating.

#### 4.2.1. Dyadic Play Session

The play session activities were designed to challenge the children's interpersonal skills and elicit dyadic friendship processes. These include conflict resolution, balance of power (equity, fairness, and aggression), affect expression (positive, negative), and intimacy (responsiveness, sensitivity), which were selected because they capture important dimensions of friendship quality for this age group [34]. Each activity was considered as a context to capture certain processes of friendship. Games that promoted peer competition might reveal assertion, aggression, control, and affect display, while games with cooperative/team rules might reveal fairness and leadership, and unstructured tasks might reveal trust and intimacy.  Table 1 illustrates the play activities and sequence.

### 4.3. Measures

*Friend Demographics and Contacts.* Demographics were collected with a 10-item form to obtain basic information including the friend's name, age, school and grade, date of birth, number of years of friendship with the target child, number of contact hours with each other, types of activities in which they engaged, and what they liked most about the friendship. The Friendship Contact Checklist [12] has fourteen common “yes/no” middle school activities (e.g., sleeping over one another's house, going to a movie together, and riding on the school bus together). The total number of shared activities is summed to indicate total contact.

*Friendship Quality.* The 40-item Friendship Quality Questionnaire [34, 35] measures the qualitative aspects of a child's very best friendship on a 5-point Likert scale. The 6 scales identified by Principal Components Analysis (PCA) matched the underlying theoretical structure with alpha coefficients of .73–.90. The scales measured the friendship characterized by: Validation and Caring (10 items; caring, support, and interest); Conflict/Betrayal (7 items; argument, disagreement, annoyance, and distrust); Conflict Resolution (3 items; the extent to which conflicts are resolved efficiently and fairly); Help and Guidance (9 items; friends' efforts to help one another with routine or challenging tasks); Companionship and Recreation (5 items; enjoyable time spent inside and outside school); Intimate Exchange (6 items; the extent of disclosure and personal feelings). Discriminate and predictive validity are reported [34]. The alpha reliability coefficients were Validation and Caring, *α* = .83, Conflict and Betrayal, *α* = .77, Conflict Resolution, *α* = .56, Help and Guidance, *α* = .82, Companionship and Recreation *α* = .56, and Intimacy Exchange, *α* = .56.

*Video Coding and Q-Sort of Play Session. *Two codings were completed to measure dyadic processes, not frequencies of behavior. After viewing the entire videotape, coders completed a Dyadic Interaction Q-Sort [12, 13, 36]. This consisted of 50-items physically displayed as cards which were sorted into seven piles according to how characteristic or uncharacteristic each item was of the dyad. Following the convention in a Q-sort, the seven piles of cards were conformed to a distribution of 5-7-8-10-8-7-5. For example, pile one consisted of 5 cards, pile two consisted of 7 cards, pile three consisted of 8 cards, pile four consisted of 10 cards, and so forth.

The coders were blind to prematurity group status and trained to a high level of agreement before coding the videotapes. Research team coders were trained by coauthor and scientist who developed the coding (J. G. Parker). The training consisted of a workshop where theoretical background instruction on each Q-sort behavior and video exemplars were introduced. This was followed by sample video segments that were viewed together, coded independently, and discussed in the group. Each coder was trained to reliability by coding an entire video play session independently. Interrater reliability was conducted by interitem correlation (>.85) between the Q-sort codes of the expert coder and training individual. During the course of the study, interrater agreement by correlation was maintained above .60 (range .60–.89). Approximately 30% of the tapes were double coded. When coders were uncertain of a code, the video was viewed by a second coder, the coding discussed, and agreement reached by consensus. Dr. Parker was available for consultation during the course of the coding period.

To check concurrent validity of the Q-sort coding, different coders rated peer-peer verbal and nonverbal behavior on a 5-point Likert scale using the Dyad Rating Scale [36]. The 15 items include disclosure (background information, attitudes and feelings, negative experiences, and gossip), mutual activities, cohesiveness, positive feelings, negative feelings, directives, boisterous play, problem behavior, leadership, and compliance. The alpha reliability coefficient for this scale was *α* = .78. Interrater reliability among coders was >90% agreement.

## 5. Statistical Analysis

The analysis had three main components matching the study goals. To answer the first goal, descriptive statistics of the dyad demographics, friendship history, and activities shared between the dyad were compared between the preterm and full term groups. For the second goal, Q-factor analysis to characterize and examine concurrent validity of the dyadic friendship behaviors, based on the Q-sort coding of the videotaped play session, was conducted according to previously developed methods [37, 38]. Using a transposed dataset (person-centered instead of variable-centered), factor loadings were obtained using PCA, and Bartlett Factor scores were saved using an eigenvalue cutoff of 1.00. Q-sort variables were then ranked in descending order by Bartlett factor scores to examine the “most characteristic” and “least characteristic” behaviors for each retained factor. PCA was conducted on the Dyad Rating Scale. Finally, analysis for the third goal involved effects of prematurity (full term versus preterm group) and gender on peer-peer friendship Q-factors and the friends' perspective of their friendship quality on the same factors. Multivariate ANOVA models were tested with peer processes predicted by prematurity and gender. Univariate analysis followed significant multivariate models.

## 6. Results

A total of 20 children, of the original sample of 186, did not participate in the play session (*N* = 166). Twelve children completed partial assessment and did not complete the play session due to scheduling conflicts. Eight participants were incapable of completing the play session due to physical and/or developmental limitations. Those unable to participate had lower IQ [*M* = 88.69, SD = 29.5 versus *M* = 98.68 SD = 15.6; *t*(171) = 2.04, *P* = .04], were in self-contained classroom [*χ* ^2^(1) = 30.2, *P* = .000], had an IEP [*χ* ^2^(1) = 9.1, *P* = .003], and special education eligibility [*χ* ^2^(1) = 11.6, *P* = .001] for physical therapy [*χ* ^2^(1) = 14.8, *P* = .000], speech therapy [*χ* ^2^(1) = 15.8, *P* = .000], and/or occupational therapy [*χ* ^2^(1) = 21.5, *P* = .000]. There were no group differences in gender [*χ* ^2^(1) = 2.1, *P* = .14], and SES [*t*(182) = 1.7, *P* = .09] between those who participated (*n* = 166) and those who did not participate in the play session (*n* = 20).

The average age for full term and preterm groups did not differ [full term, *M* = 12.2  SD = .48; preterm, *M* = 12.1  SD = .57; *t*(164) = 1.45, *P* = .15]. There were no differences between full term and preterm groups in grade level with the majority of participants in grade 7, [*χ* ^2^(5) = 9.71, *P* = .08]. As expected, the preterm group had lower birth weight [*t*(164) = 36.67, *P* = .000], gestational age [*t*(164) = 23.55, *P* = .000], greater length of hospital stay [*t*(164) = −11.94, *P* = .000], and neonatal risk [*t*(164) = −16.84, *P* = .000] compared to the full term group (see Table 2).

Results for goal 1 show that the duration of the friendship was similar (full term, *M* = 6.37 years, SD = 3.8; preterm, *M* = 5.85 years, SD = 3.8; [*t*(155) = .73, *P* = .47]), but the full term group spent more hours per week with their friend than the preterm group (full term, *M* = 23.3, SD = 15.8; preterm, *M* = 17.0  SD = 16.3; [*t*(141) = 2.03, *P* = .05]). Children born preterm were similar to the full term group in the types of contact with their friend by engaging in multiple age-appropriate activities in and outside school such as lessons, sports, visits to their homes, and sleepovers. The age and grade level of friends of children in the full term group did not differ (friend age, *M* = 12.0, SD = 1.3, *t*(156) = 1.74, *P* = .08; friend grade level, grade 7, [*χ* ^2^(9) = 16.58, *P* = .06]). Although not statistically significant, children born preterm had friends who were younger (preterm group age, *M* = 12.08, SD = .57; preterm friend age *M* = 11.5, SD = 1.5, *t*(156) = 1.74, *P* = .08). Children born preterm had more friends in a lower grade level (grade 6), [*χ* ^2^(9) = 9.71, *P* = .08].

 Most children had a same sex friend who was the same age (*M* = 11.6 years, SD = 1.5). Most children had met their friend through school, followed by the neighborhood. Most dyads were same gender pairs. There were only thirteen boy-girl dyads with most found in the preterm group and only one boy-girl dyad from the full term group. In summary, for goal 1, the descriptive characteristics of the friendship dyads full term and preterm groups were similar in duration of friendship, types of activities or contact with which they engaged, and age of friend. There were group differences in amount of weekly contact, with those born full term spending more time with their friend during the week than those born preterm. The 12-year-old children born preterm had friends in lower grade level, whereas the 12-year-old full term children had friends in the same grade level.

For goal 2, Q-sort variables were ranked in descending order by Bartlett factor scores; the highest loadings represented those play characteristics that were most like the children for that factor, and the lowest loading represented those play characteristics that were least like the children for that factor (see Table 3). In examining the most characteristic and least characteristic play behaviors, consensus was reached by the researchers and later with the statistician and developmental psychology consultants to name the factors as peer-peer friendship behavior types of leadership (asserting leadership, control, in command, assertive), distancing (disengaging behaviors, withdrawing, pulling away from dyad), and mutual playfulness (lively play, having fun together, and dyadic play in synchrony).

The analysis was repeated to ensure that the classification of play behaviors as “typical” versus “atypical” did not vary by preterm versus full term status. Results indicated no substantive differences in the most typical/atypical characteristics for the three factors for each subset of data separately. In other words, although there was a difference observed in the relative ranking of most typical behaviors for the Leadership dimension (e.g., for full term, “participant leader” was ranked as first in order of typicality, whereas for preterm, “friend complies with request” was ranked slightly higher than “participant leader”), all five of the most typical behaviors were the same for all three factors. Least typical behaviors did not vary by full term versus preterm status. For analysis on the preterm dataset only, three factors were retained, explaining 58% of the variance in the data. For analysis on the full term dataset only, three factors again were retained, explaining 57% of the variance. Therefore, the remaining analysis proceeded with the full sample.

The correlation among peer-peer behavior types shows that greater leadership characteristics were associated with less distancing (*r* = −.26, *P* < .01) and less mutual playfulness (*r* = −.41, *P* < .01). Distancing and mutual playfulness were not correlated (*r* = −09, *P* = .21).

The independently coded Dyad Rating Scale was used to examine concurrent validity of Q-factoring; the best solution of the PCA was an unrotated matrix with three components with eigenvalues >1 explaining 71.5% of the total variance. All communalities were above .50 and loadings were >.45. Nine items were loaded on the first factor labeled disclosure/cohesion, four items were loaded on the second factor labeled negative play, and two items were loaded on the third factor labeled compliance. Correlations between friendship behavior types and independent dyad ratings of play qualities supported these friendship processes. More leadership was associated with more disclosure/cohesion (*r* = .37, *P* = .000), less negative play (*r* = −67, *P* = .000), and less compliance (*r* = .12, *P* = .05). More distancing was correlated with less disclosure/cohesion (*r* = −74, *P* = .000). Mutual playfulness was correlated with disclosure/cohesion (*r* = .18, *P* = .03) and negative play (*r* = .42, *P* = .000). Taken together, these results indicate adequate concurrent validity.

Goal 3 addressed whether prematurity and gender differed between children born preterm and full term on friendship behaviors and the friend's perspective of the quality of the peer relationship. The descriptive information is first presented in Table 4 for the three friendship behavior types (Q-factors), as well as, the friend-rated friendship quality scales of validation and caring, conflict/betrayal, conflict resolution, help and guidance, companionship and recreation, and intimate exchange. In general, 12-year-old children demonstrated similar amounts of play behavior characteristics across neonatal groups. Only one significant difference was found on the friend ratings of companionship and recreation in which the full term group score was higher than the preterm group. On the examiner-rated Q-factor, a gender effect was observed, with boys engaging in play that was less characteristic of the distancing type than girls (*t*(164) = 3.27, *P* < .05). Gender effects were found for five of the six friendship quality scales with girl friends rating higher quality than boy friends in validation and caring, conflict/betrayal, help and guidance, and intimate exchange. There was no significant gender x neonatal group interaction effects.

Table 5 displays the association of Q-factors with friend ratings of friendship quality. More distancing was associated with less conflict resolution (*r* = −.17, *P* < .05) and intimate exchange (*r* = −.20, *P* < .05). More playfulness was associated with more companionship/recreation (*r* = .24, *P* < .01) and intimate exchange (*r* = .19, *P* < .05).

## 7. Discussion

With so little examined on formerly preterm children's close friendships, we focused on observation of dyadic social interactions in verbal and nonverbal behaviors with a self-identified close friend at a critically important age for friendships [1, 4]. In contrast to other methods, observation of dyadic social interactions reveals how friends act toward each other and may suggest indirect processes in the quality of peer social interaction [39]. The laboratory play session enabled measurement of nonverbal and subtle exchanges are not captured in other methods [40]. This is distinctly different from sociometric measurement and parent report or adolescent report methods of social function seen in preterm follow-up studies [23, 41]. We do not know whether the number of friends or size of the peer social network is smaller for adolescents born preterm as this was not the focus of the study.

Twenty children, 11% of our sample, were unable to participate in the play session due to scheduling conflicts, or intellectual, learning, or motor disability. Most of this developmental disability was mild or moderate as there were few children (*n* = 8) with severe medical complications such as cerebral palsy, blindness, or neurosensory hearing loss (<10% of our cohort). Taken together with those who were able to participate in the play session but had younger aged friends in a lower grade, this suggests that prematurity does affect later social relationships at early adolescence for a small percent of formerly preterm infants.

The first goal of the study was to describe demographic characteristics of close friends at age 12 in children born preterm. Of the formerly preterm children who were able to participate, most had an acknowledged close friend who was not necessarily tied to boundaries of school. Many had friendship with an older or younger peer outside of school. This suggests that although formerly preterm children may need academic support in school, they do have at least one quality friendship outside of school. From a cognitive processing theoretical perspective, these findings suggest that, for the most part, children born preterm do have a close peer relationship and may be indicative that they are able to accurately interpret the verbal and nonverbal behaviors.

One explanation as to why most children born prematurely had a close peer relationship may be attributed to the sources of support or assistance in this process. For example, parents of children born preterm tend to be overprotective due to the fragile health of their preterm child. This “overprotective” quality may place the parent in the role of initiating more invitations for peer contact than parent of children born full term. This, however, may result in diminished opportunities for children born preterm to learn the requisite cues during social interaction [21, 23, 24].

In goal 2, the factors of leadership, distancing, and mutual playfulness characterized dyadic friendship behaviors. The leadership friendship type could suggest aggression or a defensive behavior learned from peer group experiences. A close review of the items does not include aggressive behaviors (verbal aggression, power assertion) or defensive behaviors (see Table 3), as these Q-sort items did not load on this factor. This was an unexpected finding given reports that cognitive problems, hyperactivity, and behavioral problems in preterm samples may contribute to inaccurate reading of peer cues [22, 31, 32]. It is possible, that the nature of the laboratory play session, which required the target child to extend an invitation, placed the preterm child in a “leadership” role which was carried throughout the five play activities. It is also possible that exhibiting leadership behavior was a learned defensive, yet protective approach within the close friendship dyad.

In the distancing friendship type, the target child is focused on task rather than social interchange. Minimal, if any, disclosure, story-telling, or sharing exists. These behaviors suggest that the target child actively disengages from their friend during play. This finding is contrary to behaviors expected between two close friends, especially given the importance of peer friendships at adolescence [2]. High-quality friendships have positive features including disclosure of personal thoughts and feelings, intimacy, loyalty, and support as well as negative features of conflict, rivalry, and dominance attempts [39]. Instead, the dyads may have developed a negative interaction repertoire that has become routine engendering negative reactions from peers. Shyness/withdrawal, a similar concept to distancing, was related to peer rejection [42]. Perhaps distancing is a protective mechanism for these children when faced with challenges in front of competent peers. Alternatively, it could be due to peer social experiences of rejection or bullying that has been reported with formerly preterm children [23]. If friendships during early adolescence of children born prematurely are characterized by distancing and little intimacy coupled with the decreased time with family, increased time alone and less positive affect increased risk for internalizing problems such as depression may develop [1, 10]. Internalizing problems of depression, anxiety, and withdrawal have been reported in international follow-up studies of preterm infants [10, 43].

Within the comfort of a close friendship, good-natured interactive behavior identified as mutual playfulness was observed. The Q-sort items for this friendship type are clearly dyadic enjoyment. Reciprocity or mutuality is a basic element of friendships from childhood to adulthood, and revolves around the sharing of common activities in adolescence [7]. But mutuality at this age also involves self-disclosure and trust. In considering both findings of distancing and mutual playfulness friendship types, the latter may be a “surface structure” rather than a “deep structure” as described by Hartup. Surface structures “refer to the social exchanges that characterize them” whereas deep structures refer to “the social meaning (essence) of relationships” [7]. The mutual playfulness seen among these dyads may be an indication of age-appropriate social repertoire that is unstable in the long-term.

Contrary to our hypothesis and notably absent from the three peer friendship types found in this study was intimacy. Close personal friendships demand higher expectations from each child and distinctive personal processes (e.g., affection, intimacy) that differ from a peer group [12]. Friendship quality, characterized by intimacy and prosocial behavior, mainly affects children's success in the social world of peers [39]. Close and good friendships may improve positive contacts with other peers and lead to a larger circle of friends. This finding is not likely to be related to the play session methodology used in the study since global intimacy was observed using a similar laboratory play session during the snack activity among abused and nonabused dyads and in another study of triads [12, 13]. In addition, the levels of intimate exchange rated by the friend in this study are comparable to other samples [34]. Thus, there is support for the unexpected finding.

Generally, friendship interaction behaviors influence whether a child is accepted by peers, but there are other characteristics (e.g., physical appearance, social class) that come into play. Interpersonal dynamics change with larger groups, although studies have shown that children who are better accepted by a group are likely to have a best friend [34].

Related to goal 3 and as hypothesized, gender effects, not specific to prematurity, were found. The gender differences where boy dyads reported more distancing and girls reported higher validation and support, help and guidance, conflict resolution, intimate exchange but did not differ from boys on conflict and betrayal, companionship and recreation have been reported [13, 34]. Girls tend to self-disclose, be more supportive, and have more conversation than boys [13]. More recently, corumination has been described in adolescent girls who have detailed, exhaustive discussions of problems with friends [44]. High concern about the social self in positive quality friendships may exacerbate internalizing problems [33]. The friendship quality data represents only one view of the friendship, the friends' perception. We do not know whether their view was shared by the both children in the dyad.

Our longitudinal analysis shows little prediction from the events of preterm birth. In many ways, our findings reflect those found in studies with healthy samples of school-age children. Given our cross-sectional findings of friendship processes and the numerous reports of the developmental limitations of formerly preterm children, one unexamined consideration is whether their social interaction ability can enhance competence in other areas of development. For example, school learning takes place in a social environment and preterm children often have learning difficulties. Identification of close friendship behaviors better elucidates characteristic processes that are important to peer group affiliation and other areas of competence. Promoting positive relationship behaviors could enhance academic achievement, perhaps as a mediator to later outcomes.

 This study offers new insights into the friendship behaviors of at early adolescence, a time when peer friendships increase in importance. There were low alpha coefficients for three scales on the Friendship Quality Questionnaire (conflict resolution, companionship and recreation, intimacy exchange). Even though there is some ambiguity about acceptable alpha coefficients, it is generally agreed that internal consistency estimates should be ≥.70 [45]. The Friendship Quality Questionnaire was used to explore the relationships between friend's rating of quality and the three friendship types and was not a key measure in the study. Given that there is so little known about peer-peer friendship behaviors, these findings should be interpreted as exploratory.

Prematurity is a rising national public health problem and economic burden [46]. Of the 4 million infants born in the US, 12.5% were premature, an 18% increase since 1990 [47]. Up to 50% of premature infants have later dysfunctions, such as learning disabilities, attention problems, cognitive deficits, neuropsychological deficits, and behavioral problems [48]. With national concern for preterm infant outcomes, the prospective, longitudinal findings of this study provide an initial view into the social-behavior processing of early adolescent peers.

## Figures and Tables

**Table 1:** Sequence of dyad play session activities.

Activity	Aim	Dyadic processes	Number of trials and total time
*1. Modified perfection * [12]	To fit as many pieces as possible into the corresponding matrix holes before clicking timer stopped and “popped” the pieces from the spring loaded board. The more pieces fitted within 45 seconds, the more points earned. If the board popped, no points were earned.	The dyad decide when to stop the timer by pushing a switch before the platform popped, thus earning points. *Observed*: playfulness and humor, negotiation, and willingness to compete against each other.	4 trials; 4 minutes (2 trials-dyad worked together for equal points; 2 trials-dyad played individually)

*2. Nintendo 64 “diddy kong racing”*	Dyad raced each other to the finish line using steering wheel controllers. Encouraged to capture “bananas” along the race routes to earn points. Each player could simultaneously play and view the other's progress on a split screen.	Dyad decide whether to individually earn points or pool their racing “bananas” for both players to earn 1 point and/or earn 5 points by crossing the finish line. This was a “Prisoner's Dilemma” or bargaining task. *Observed*: competition, cooperation, attention to task versus friend, affect intensity, and conversation about the game.	2 trials, 13 minutes (1 trial practice; 1 trial race competition)

*3. Modified connect 4*	With a set of red, black, and white checkers, and the vertical plastic grid, the dyad took turns to make “connect 4” or letter the “T”. Points earned: 2 points for each Connect Four of their own; 3 points for each “T” made together with the white checkers.	Forced choice activity in which dyad must decide whether to play by taking turns and connecting four red or black checker pieces vertically, horizontally, or diagonally or to work cooperatively with their partner to make the letter “T” with the white checkers. *Observed*: negotiation, competition, conflict, and whether the dyad had equal influence or authority over the other.	2 trials, 10 minutes (dyadic choice made at start of each trial)

*4. Puzzle task* [13]	A challenging 100-piece jigsaw puzzle to complete by dyad within short time. Dyad told that only a few twosomes, listed on a winner's chart, had completed the puzzle within the time. They were challenged to beat the record. Points earned: 2 points for both players and their names added to the “winner's chart”.	*Observed*: cooperation, pursuit of common goal, leadership, division of power, and ability to sustain task focus.	1 trial, 12 minutes

*5. Share a snack* [12, 13]	Dyad invited to make and share ice cream sundaes, and then cleanup. A small cart contained bowls, spoons, ice cream, sprinkles, whipped cream, and chocolate sauce was provided. Later cleaning supplies were given.	*Observed*: casual, unstructured conversation, social behaviors (serving a friend, social talk, and gossip), intimacy of the friendship, willingness to self-disclose, recounting of shared experiences, and initiative or teamwork in cleanup.	1 trial, 12 minutes

**Table 2:** Neonatal characteristics for study sample by prematurity group (*N* = 166).

	Full term ≥38 wga *n* = 40 *M* (SD)	Preterm <37 wga *n* = 126 *M* (SD)
Boys/girls (*n*)	19/21	54/72
Birth weight* (grams)	3422.9 (338.6)	1257.2 (321.2)
Gestational age* (weeks)	39.9 (.81)	29.9 (2.6)
Length of stay* (days)	3.0 (.42)	52.3 (26.0)
Hobel neonatal risk score* [14]	1.5 (3.6)	85.9 (31.5)
Bronchopulmonary dysplasia* (*n*/%)	0 (0%)	22 (17%)
Sepsis^∧^ (*n*/%)	0 (0%)	12 (10%)
Necrotizing enterocolitis^#^ (*n*/%)	0 (0%)	16 (13%)
Meningitis (*n*/%)	0 (0%)	3 (2%)
Intraventricular hemorrhage (*n*/%)	0 (0%)	25 (20%)
SES [15]	39.4 (12.5)	42.3 (14.6)

Note. wga: weeks of gestational age; ^∧^ *P* = .05; ^#^ *P* = .01; *P = .000; SES: socioeconomic status.

**Table 3:** Q-factoring results: examples of most and least characteristic qualities of peer-peer friendship processes.

	Leadership	Distancing	Mutual playfulness
Most characteristic qualities	Target child is leader	Target child more interested in activity than friend	Dyad reaches agreement easily
	Friend complies with requests	Target child is cautious	Dyad shares
	Dyad is coordinated	Dyad engages in individual play	Dyad plays happily
	Dyad happily plays	Dyad is impolite or rude	Dyad verbal negotiation is equitable
	Target child is direct and assertive	Target child appears disjointed in play	Target child expresses enjoyment verbally

Least characteristic qualities	Dyad engages in individual play	Target child shares gossip	Target child controls
	Friend ignores target child's suggestions	Target child self-discloses	Target child criticizes
	Target child complies with friend requests	Dyad shares secrets	Dyad uses threats/aggression
	Target child wanders or is bored	Target child tells positive stories	Dyad is wild, out of control
	Target child self-discloses	Target child shares personal facts and information	Dyad is impolite, rude

Note. “Target child” refers to research study participant; “friend” refers to study participant's invited friend.

**Table 4:** Mean scores of friendship behavior types and friendship quality scales at age 12 by prematurity group.

	Full term ≥38 wga *n* = 40 *M* (SD)	Preterm <37 wga *n* = 126 *M* (SD)
Friendship behavior types^a^		
Leadership	.38 (.46)	.50 (.39)
Distancing^b^	.12 (.27)	.05 (.32)
Mutual playfulness	.15 (.29)	.07 (.26)
Friendship quality scales		
Validation and caring^c ^	32.16 (7.15)	32.31 (6.35)
Conflict resolution^c^	8.88 (3.09)	9.04 (2.96)
Conflict/betrayal^c^	17.43 (3.26)	18.14 (2.64)
Help and guidance^c^	26.50 (7.10)	24.80 (6.70)
Companionship* *and* *recreation^*∧*^	12.77 (4.42)	11.07 (4.22)
Intimate exchange^c^	17.05 (5.22)	15.59 (6.19)

Note. ^∧^ *P* = .04; ^a^average factor loading, range −1  to  1. ^b^boys > girls (*P* = .002); ^c^girls > boys.

**Table 5:** Pearson correlations: associations among adolescent friendship behavior types and friend-rated friendship qualities scales.

	1	2	3	4	5	6	7	8
(1) Leadership	1							
(2) Distancing	−.26±	1						
(3) Mutual playfulness	−.41±	−.10	1					
(4) Validation and caring	.10	−.16	.14	1				
(5) Conflict resolution	.04	−.17*	.10	.63±	1			
(6) Conflict/betrayal	.01	−.04	.10	.29±	.29±	1		
(7) Help and guidance	.00	.01	.13	.71±	.53±	.145	1	
(8) Companionship and recreation	−.05	−.05	.24±	.31±	.27±	.06	.42±	1
(9) Intimate exchange	.06	-.20*	.19*	.70±	.51±	.16*	.63±	.49±

Note. ± 0.01 level (2-tailed). *0.05 level (2-tailed).
